# Occurrence of *Clostridium difficile* in seasoned hamburgers and seven processing plants in Iran

**DOI:** 10.1186/s12866-014-0283-6

**Published:** 2014-11-25

**Authors:** Zahra Esfandiari, Scott Weese, Hamid Ezzatpanah, Mohammad Jalali, Mohammad Chamani

**Affiliations:** Department of Food Science and Technology, Science and Research Branch, Islamic Azad University, Tehran, Iran; Department of Pathobiology and Centre for Public Health and Zoonoses, Ontario Veterinary College, University of Guelph, Guelph, Ont N1G2W1 Canada; Department of Animal Science, Faculty of Agriculture and Natural Resources, Tehran Science and Research Branch, Islamic Azad University, Tehran, Iran

**Keywords:** *Clostridium difficile*, Meat, Processing plants, Ribotyping

## Abstract

**Background:**

The recent increment of the incidence of Community Associated *Clostridium difficile* Infection (CA)-CDI has led to speculation that this disease is associated to foodborne transmission. Therefore it is critical to establish the community sources of CDI in order to implement the appropriate interventions. The present study was conducted to evaluate the prevalence of *C. difficile* in seasoned hamburger and examine the sources of *C. difficile* dispersal in hamburger processing plants. A total of 211 samples including hamburger ingredients, the final product, processing equipment and food contact surfaces were collected from seven hamburger processing plants to evaluate the routes of dispersal of *C. difficile*. The samples were assessed for the occurrence of *C. difficile* using culture and polymerase chain reaction (PCR) methods. All isolates were screened for the existence of toxin A, B and binary toxin genes. In addition, isolates were subjected to PCR ribotyping.

**Results:**

Overall, 9/211 (4.2%) samples were positive. Toxigenic *C. difficile* were detected from 2/7 (28.5%) hamburger processing plants, in (3/54) 5.6% of beef meat samples, (2/56) 3.5% of swabs taken from the environment and (4/56) 7.1% of hamburger samples after both molding and freezing. *C. difficile* was not found in 45 non-meat ingredients including 14 defrosted onions, 14 textured soy proteins and 17 seasonings. All isolates contained *tcd*B gene while 7 strains were positive for *tcd*A and two remaining strains were negative for *tcd*A. None of the isolates harbored binary toxin gene (*cdt*B). PCR ribotyping of 9 isolates categorized into four ribotypes (IR21, IR 22, IR 23 and IR24). Ribotype IR 22 was the most common type 6/9 (66.6%) found. This genotype was isolated from raw meat, environmental samples and hamburger after both forming and freezing in one processing plant, suggesting raw beef meat as a possible major source of contamination.

**Conclusions:**

Hyper-virulent strains of ribotype were not found in this study however, occurrence of other toxicgenic strains indicate the public health significance of contamination of this product.

## Background

*Clostridium difficile* is an anaerobic Gram-positive endospore forming bacterium that is considered to be the most significant cause of nosocomial infection acquired in humans (CDI) [1]. To date, the most significant epidemic strain of *C. difficile* encountered in healthcare facilities within North America and Europe has been a strain classified by restriction enzyme analysis are BI, pulsed field gel electrophoresis (PFGE) as NAP1 and PCR ribotyping as ribotype 027 (B1/NAP-1/027) [2]. This strain is widely disseminated internationally and as a common cause of both endemic and epidemic CDI. In addition, recent increases in community associated (CA)-CDI in patients with no recent contact with clinical environments has led to speculation of other potential sources of exposure. The potential that CDI could be a foodborne disease has been raised based on variable but often high rates of *C. difficile* colonization of food animals and identification of *C. difficile* in retail meat products [3-9]. Additionally, ribotype 078, a strain commonly associated with CA-CDI, is a predominant strain in food animals and food heightening concerns of a possible food origin [4,10].

Data regarding the contamination of food of animal origin are mostly reported from Europe and North America. These studies have focused mostly on the prevalence of *C. difficile* at the farm or retail level, and only a few surveys have been performed at meat processing plants [6,7,11-13]. Rodriguez-Palacios et al (2007) reported the presence of *C. difficile* up to 20% in unseasoned hamburgers in Canada [3]. Further studies indicated that *C. difficile* spores are relatively unaffected by processing such as freezing, refrigeration and cooking ([14-16]. Furthermore, the epidemiology and prevalence of *C. difficile* in food in Iran are limited, despite the importance of CDI and a previous study that found ribotype 078 to be a leading cause of CDI in people at one hospital in Isfahan, Iran [17]. The rate of CA-CDI reported to be 24% in Iran, with no data available on sources of infection [17].

Therefore, the objective of the present study was to determine the prevalence of *C. difficile* in seasoned hamburger and examine the sources of *C. difficile* dispersal in hamburger processing plants in the same location in Iran.

## Methods

### Sampling design

**Figure 1:**
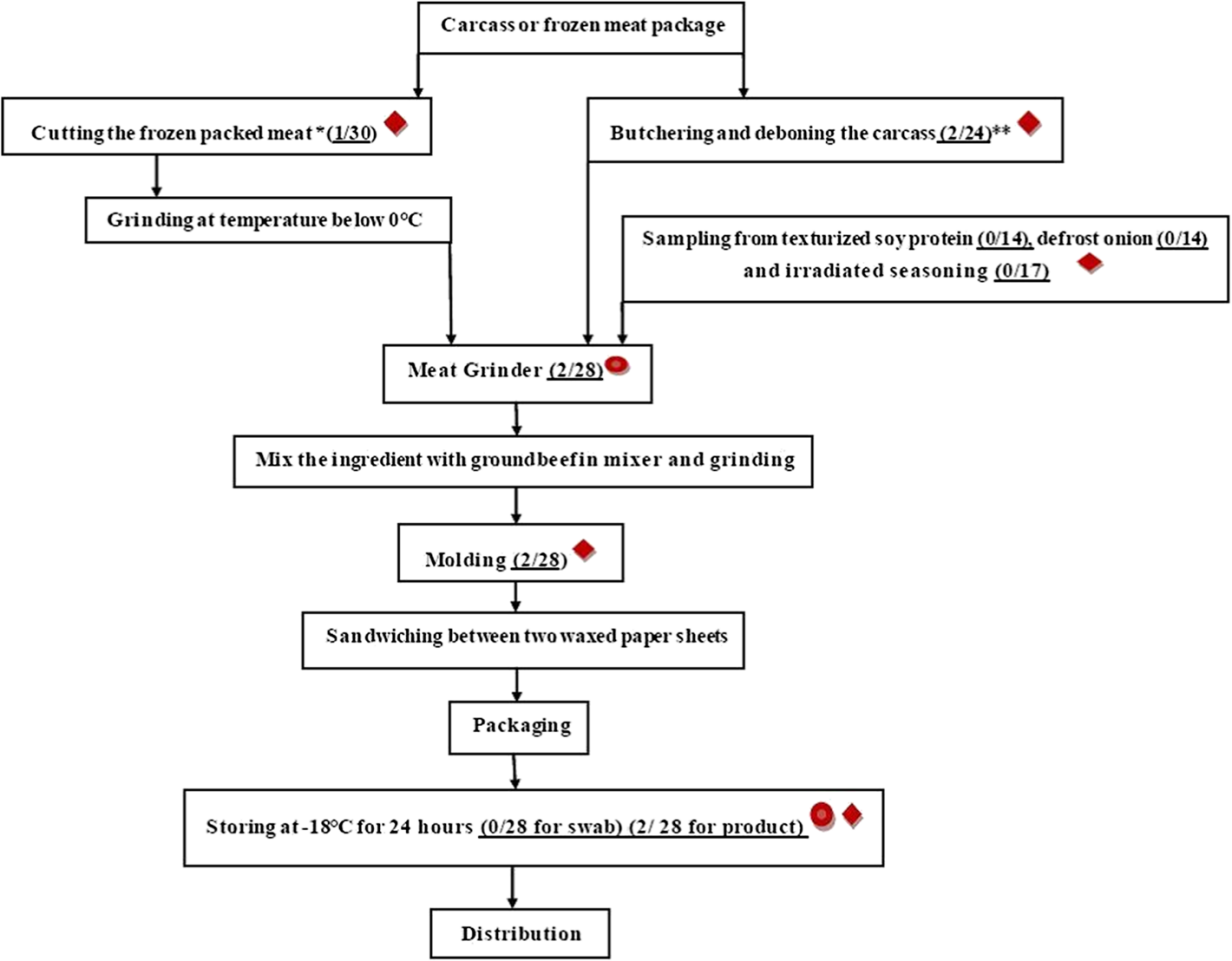
**Flow diagram for the production of hamburgers with sampling points (red diamonds for ingredient/final product and red circles for swab samples).** *Packed boneless meat imported from Brazil. **Carcass meat provided from slaughterhouses in Iran (Number of positive samples for *C. difficile* in total sample is mentioned in parenthesis in related box).

This study was conducted in Isfahan, central part of Iran (subtropical zone; mean long time rainfall, 120 mm; mean long time temperature, 33°C and 17.6°C in summer and autumn; altitude, 1,555 m; longitude, 51°30′E; and latitude, 32°31′N). Samples were taken from seven (coded A to G) available hamburger processing plants that supply products nationwide from July to December 2012. Samples collected during four visits (two visits in summer and two visits in autumn). Three of seven processing plants (coded A, B and G) were Hazard Analysis Critical Control Points (HACCP) certified. However, all processing plants were ranked in terms of Quality Assurance Managements (QA) and implementation of Good Manufacturing Practice (GMP) by the Ministry of Health (Category I, II and II). The hamburger patties consisted of 35% texturized soy protein, 30% beef meat, 16% onion, 9% frying oil, 8% wheat flour, 1.7% salt and 0.3% irradiated seasonings. The ground product was molded, sandwiched between two waxed paper sheets, packaged and frozen at −18°C for 24 h (Figure 1). Samples were collected based on their production day per week (one or two day production per week). At the hamburger processing plants, the ingredients had a different expiry dates and the irradiated seasonings, texturized soy protein and frozen onion, kept for longer time (for more than a few months). Therefore these products sampled less frequently than the beef meat (normally supplied on weekly bases) as a main ingredient of hamburger.

A total of 211 samples were obtained including: beef meat (n = 54), textured soy protein (n = 14) irradiated seasoning (n = 17), defrosted onion (n = 14), hamburger patties after molding (n = 28) and hamburger patties after freezing (n = 28). Approximately 5-10 gram of each sample were collected aseptically in sterile bottles and transported in an insulated cold box to the laboratory. In addition, 56 environmental samples were taken from meat grinder and the freezer chamber wall. Cotton swabs moistened with sterile 0.85% NaCl were used to collect samples from approximately 20 cm^2^ areas during working hours. All samples were analyzed on the day of sampling at the Infectious Diseases and Tropical Medicine Research Centre, Isfahan University of Medical Sciences, Isfahan, Iran.

### Isolation of *C. difficile*

For culture analysis, a method of Rodriguez-Palacios *et al*. [3] was used. Briefly, 5 g of sample or swabs was added to 25 ml of selective enrichment *C. difficile* broth (1 liter containing 40 g proteose peptone, 5 g disodium hydrogen phosphate, 1 g potassium dihydrogen phosphate, 0.1 g magnesium sulfate, 2 g sodium chloride, 6 g fructose and 1 g sodium taurocholate supplemented with 500 mg cysteine hydrochloride, 12 mg norfloxacin and 32 mg moxalactam) in 50 ml falcon tubes and incubated for five to seven days at 37°C. For each sample, an alcohol shock to kill the vegetative cells was performed by adding a volume of 2 ml of enriched broth to an equal volume of absolute ethanol in a centrifuge tube, gently vortexed and kept at room temperature for 2 h. The samples were centrifuged at 10000 × g for 10 min, after which the supernatant was removed. The pellet was streaked onto *Clostridium difficile* Moxalactam Norfloxacin agar medium (CDMN agar) with 7% sheep blood and anaerobically incubated for 48 h at 37°C using an Anoxomat system (MART Microbiology B.V., Drachten, Netherlands). Isolates were presumptively identified as *C. difficile* by morphology, cresol/horse odor and L-proline β-naphthylamide disk (Prodisk, Hardy Diagnostics, Santa Maria, CA, USA). Culture and molecular assay were performed in a separate laboratory and negative controls were used in the entire process.

### Molecular characterization of isolates

Suspected colonies were sub-cultured onto blood agar plates and incubated anaerobically at 37°C/24 h. DNA extraction was performed by transferring 3-5 colonies into 100 μl of sterile distilled water, heating at 95°C for 3 min and then centrifuged at 7500 × g for 15 min. The supernatant was used as a DNA-template PCR detection of genes encoding triose phosphate isomerase (*tpi*), toxin A and B (*tcd A* and *tcd B,* respectively) and binary toxin (*cdtB*) as described by Lemee *et al*. [18]; Stubbs *et al*. [19]. Isolates were also subjected to PCR-ribotyping as described by Bidet *et al*. [20]. Interpretation of ribotyping results was performed by visual identification. Ribotype patterns were designated (i.e. IR22) by internal nomenclature. A reference strain of ribotype 027 was available for comparison.

## Results and discussion

*C. difficile* was isolated from 9/211 samples (4.2%; 95% CI [1,7]) from 2/7 (29%) processing plants (Table 1). These two processing plants (C and D) did not hold HACCP certification and were ranked in lowest level (category III) in terms of QAM and GMP. This is unsurprising because *C. difficile* contamination would be from carcass contamination by feces or intestinal contents, something that HACCP practices are designed to minimize. Similarly, lower rates of contamination of *C. difficile* in slaughterhouses have been reported when HACCP principles were implemented [11].

**Table 1:** **Occurrence of** ***C. difficile*** **in hamburger ingredients, hamburger and environmental samples within the hamburger processing plants**

**# of** ***C. difficile*** **positive (%)/# of samples**								
**Processing plant**	**Raw meat**	**Textured soy protein**	**Seasoning**	**Onion**	**Swab**	**Hamburger after molding**	**Hamburger after freezing**	**Total**
A	0/8 (0)	0/2 (0)	0/3 (0)	0/2 (0)	0/8 (0)	0/4 (0)	0/4 (0)	0/31 (0)
B	0/6 (0)	0/2 (0)	0/3 (0)	0/2 (0)	0/8 (0)	0/4 (0)	0/4 (0)	0/29 (0)
C	1/8 (12.5%)	0/2 (0)	0/1 (0)	0/2 (0)	0/8 (0)	0/4 (0)	0/4 (0)	1/29 (3.4)
D	2/8 (25%)	0/2 (0)	0/4 (0)	0/2 (0)	2/8 (25%)	2/4 (50)	2/4 (50)	8/32 (25)
E	0/8 (0)	0/2 (0)	0/2 (0)	0/2 (0)	0/8 (0)	0/4 (0)	0/4 (0)	0/30 (0)
F	0/8 (0)	0/2 (0)	0/2 (0)	0/2 (0)	0/8 (0)	0/4 (0)	0/4 (0)	0/30 (0)
G	0/8 (0)	0/2 (0)	0/2 (0)	0/2 (0)	0/8 (0)	0/4 (0)	0/4 (0)	0/30 (0)
Total	3/54 (5.6)	0/14 (0)	0/17 (0)	0/14 (0)	2/56 (3.5)	2/28 (7.1)	2/28 (7.1)	9/211 (4.2)

Raw beef meat was the only ingredient found to be contaminated with *C. difficile* in 3/54 (5.6%; 95% CI [1,15]).

Detection of *C. difficile* in raw beef was unsurprising and demonstrates the potential of meat as a source of *C. difficile* dispersal, something that is logical given its presence in food animals. However, *C. difficile* has been found in vegetables and other food items like fish, shellfish, edible bivalve molluscs, egg, ready to eat food (RTF) and salads [4,21-26], so while *C. difficile* is likely always of human or animal origin, it is important not to ignore the various potential sources that might have been contaminated by human or animal sources (e.g. manure contamination of water, indirect contamination by hands). If the main source of *C. difficile* contamination of hamburger is raw beef and this bacterium is endemic in the cattle population, it is likely that this organism can be introduced continuously.

The relatively low prevalence of *C. difficile* reported in the present study (5.6%) is in the range of recently published studies from numerous countries reporting the isolation of *C. difficile* from raw beef spanning 1.65 to 42.4% of samples collected at retail level [4,5,9,27,28]. Previous Iranian data are limited, with a report of contamination of 1.65% (2/121 samples) raw beef samples (9) and 2.8% (1/35) beef samples in meat packaging plants [29]. In contrast, this organism was not isolated from any of 145 raw chopped beef samples in the Netherlands [30].

Three different ribotypes were identified in raw beef (Table 2), with one accounting for 6/9 (67%) isolates. This strain was only found at one plant, but it was identified at two different timepoints. It is interesting to note that 1 out of 3 *C. difficile* strains was recovered from meat imported from Brazil. This strain had a distinct ribotype IR 21 pattern and was the only isolate obtained from processing plant C. The other contaminated beef samples originated from processing plant D. In both samples the meat was supplied from the same Iranian slaughterhouse but with different slaughtering dates. The commonness of one strain is not surprising if one (or a small number) of strains predominate in the Iranian cattle population, as is the case with ribotype 078 in many western countries. There is inadequate information about the *C. difficile* population structure in Iranian cattle to put these results into context. Another consideration would be cross contamination within the facility, resulting in numerous positive samples from one source. Laboratory contamination cannot be dismissed, but contamination of multiple samples from just one facility when samples from other facilities were being processed in parallel would be unlikely.

**Table 2:** **Molecular characterization** ***C. difficile*** **strains isolated from hamburger and its ingredients in processing plants**

**Source of isolation**	**Hamburger processing plant**	**Toxin gene profile**	**Ribotype**	**Sampling date**
Raw meat	C	*tcA* ^*-*^ *tcdB* ^*+*^ *cdtB* ^*-*^	IR21	17^th^ November
Raw meat	D	*tcdA* ^*+*^ *tcdB* ^*+*^ *cdtB* ^*-*^	IR22	4^th^ September
Swab from grinder	D	*tcdA* ^*+*^ *tcdB* ^*+*^ *cdtB* ^*-*^	IR22	4^th^ September
Hamburger after molding	D	*tcdA* ^*+*^ *tcdB* ^*+*^ *cdtB* ^*-*^	IR22	4^th^ September
Hamburger after freezing	D	*tcdA* ^*+*^ *tcdB* ^*+*^ *cdtB* ^*-*^	IR22	4^th^ September
Raw meat	D	*tcdA* ^*+*^ *tcdB* ^*+*^ *cdtB* ^*-*^	IR23	17^th^ October
Swab from grinder	D	*tcA* ^*-*^ *tcdB* ^*+*^ *cdtB* ^*-*^	IR24	17^th^ October
Hamburger after molding	D	*tcdA* ^*+*^ *tcdB* ^*+*^ *cdtB* ^*-*^	IR22	26^th^ November
Hamburger after freezing	D	*tcdA* ^*+*^ *tcdB* ^*+*^ *cdtB* ^*-*^	IR22	26^th^ November

*C. difficile* was not found in any of the 45 non-meat ingredients of hamburger (14 textured soy proteins, 17 seasoning and 14 defrosted onions). Various potential explanations can be hypothesized. One is that the raw ingredients for these materials may be less likely to be contaminated. Another is that processing might have eliminated any *C. difficile* contamination, such as the heat generated during extrusion of textured soy protein [31].

The seasoning samples collected in this study were a mixture of black pepper, cinnamon, sumac and cumin were free of *C. difficile*. Seasonings could be contaminated with spores of *Clostridium* species because of the lack of proper sanitary conditions during collection or as a consequence of open air drying procedures [32]. However, in the processing plants selected in this study, the seasonings were supplied to the meat processing plants after being sterilized by irradiation in 2 KGy. A dose limit of irradiation for decontamination of microbial spores is in the range of 1-4 KGy [33], so the levels used here could have inactivated any *C. difficile* spores that might have been present.

Failure to isolate *C. difficile* from onions was not particularly surprising given the small sample size, but this bacterium has been isolated from onions in UK [34]. Similarly *C. difficile* was not found in a small number of onions in a Canadian study [22].

*C. difficile* spores are environmentally tolerant and resistant to many disinfectants, so it is not surprising to have found contamination in 2 out of 56 of environmental sites (3.5%; 95% CI [0, 12]). The two strains that were found isolated from one processing plant (D) during two visits approximately 6 months apart, although it is unclear whether this represents long-term persistence or (more likely) repeated contamination. Regardless, these results suggest that the hygiene procedures for cleaning and sanitation were not adequate. The standard operation procedure for sanitation used in processing plant D was washing the grinder with peracetic acid solution (4%) then rinsing with warm potable water. In the other plants where no *C. difficile* was found, the procedure was washing with sodium hydroxide solution (5%) followed by peracetic acid (4%) washing. The usage of oxidative agents and acids such as hypochlorite, hydrogen peroxide and peracetic acid is recommended to break the chemical bonds of food soils that build up the biofilm [35]. It has also pointed out that any residual organic material involved in biofilm formation could facilitate the attachment of spores and vegetative cells to meat processed in a grinder [11].

Of 28 hamburger samples taken at each step after molding and freezing, 4 (7.1%) were positive for *C. difficile*. As all positive isolates of *C. difficile* found in the same meat processing plant (D) belonged to an identical clone (RT 22), which was found on two separate occasions. This could suggest a common source of contamination, although inadequate data are available about strains found in food animals in Iran to properly interpret this finding. If this is a predominant food animal strain, the strain distribution noted here could simply reflect the background contamination of incoming meat products, although that would not explain the discrepancies in prevalence between facilities. Therefore, there must be concern that detection of the identical genotype (RT 22) from raw meat and final product at the same processing plant may indicate the persistence of this genotype during processing. In addition, colonization of the identical genotype in this processing plant may indicate the ineffectiveness of cleaning and sanitation. Complementary typing method such as multilocus variable-number tandem-repeat analysis (MLVA) or PFGE are needed to further investigate the persistence of *C. difficile* in the environment [36].

Detection of *C. difficile* from hamburger patties in 2/28 (7.1%; 95% CI [0, 23]) after 24 h freezing at – 20°C indicates the survival of spores at freezing temperature, something that is well established in human fecal samples [14]. However there are no data on effect of freezing on survival of spores in meat.

The results of molecular characterizing of the 9 *C. difficile* strains are presented in Table 2. All isolates contained *tcd*B while 7 isolates from 2 strains also possessed *tcd*A. Interestingly, all isolates lacked *cdtB.* This was unexpected given the high prevalence of binary toxin positive strains reported in other studies of raw meat, the predominance of ribotype 078 (a *cdt* possessing strain) in food animals in various countries and the commonness of *cdtB* in human isolates from a recent study [6,9,13]. These results indicate that further study of the strain distribution of *C. difficile* in animals and humans in the region is required.

PCR ribotyping of 9 isolates categorized into four ribotypes (IR 21, IR22, IR23 and IR24) (Table 2). Ribotype IR 22 was the most frequently 6/9 (66.6%) encountered in our study. This genotype isolated from raw meat, environmental samples and hamburger both after forming and freezing in one processing plant (D), suggests that raw meat may be the major source of contamination. However the specific source of *C. difficile* in meat needs to be established. The gastrointestinal tract is the most important source of *C. difficile* contamination [9].Transmission through animals’ hides, the slaughterhouse environment, the processing facility environment, processing equipment and the hands of personnel handling meat, must also be considered [9].

Inactivation of *C. difficile* spores by most of the cleaning and sanitation practices is difficult; therefore its accumulation in the environment increases the possibilities for contamination of meat. Ribotype IR 22 was isolated from various stages in processing plant D in both visits in the summer and autumn suggesting the excellent survival of spores in the processing plant. Ribotypes IR23 and IR24 were also found in processing plant D suggesting a relatively higher genetic diversity among *C. difficile* in this processing plant. Ribotype IR21 was the only distinct genotype found in beef meat imported from Brazil indicating the possibility of a geographical relationship of the genotypes.

Recently, hyper-virulent PCR ribotype 027 has emerged in North America and Europe in links to the hospital outbreaks [2]. This strain has not been reported yet in Iran in either food or clinical samples [9,17], potentially due to the lack of sufficient research in this area. Ribotype 027 also was not found in the present study, nor was ribotype 078, based on inference of the lack of *cdtB* in any isolate. We have previously reported ribotype 078 as a common strain in both humans and meat in Iran [9,17], so the absence of this strain in the current study was surprising.

This study is subjected to some limitations. First, sampling were conduced based on expiry date of ingredients where non-meat products kept for a significantly longer times. Therefore these products sampled less frequently than meat, resulting unbalanced sampling. Second limitation of this study is that only ribotyping used as strains characterization. Complementary typing methods such as MLVA or ideally whole genome sequencing would be needed to further investigate the epidemiology of *C. difficile* in the hamburger production facilities. Third, the possibility of lab cross-contamination of samples cannot be dismissed, although it may be unlikely for reasons cited above.

## Conclusions

In conclusion, the study demonstrated the existence of toxigenic *C. difficile* in hamburger processing plants through different potential reservoirs such as raw meat and facilities. Occurrence of *C. difficile* in hamburger as a commonly consumed food in Iran is of public health significance although hyper-virulent strains of ribotype 027 and 078 were not found in this study. For reduction or prevention of *C. difficile* prevalence in food products, Good Manufacturing practices (GMP) and Hazard Analysis Critical Control Point (HACCP) system should be applied in food industries and followed by post-production control procedures by consumers such as proper cooking based on the adequate time for destruction of C*. difficile* spores [37]. Susceptible individuals with increased risk for development of CA-CDI should be also educated to minimize the exposure to this pathogen in the food supply [37].

## Abbreviations

*C. difficile*: *Clostridium difficile*
CDI: *C. difficile* infection
CA-CDI: Community associated- *C. difficile* infection
PCR: Polymerase chain reaction
